# Supplementary material for: Non-calorimetric determination of absorbed power during magnetic nanoparticle based hyperthermia
Source: Sci Rep. 2018 Aug 23;8:12667. doi: 10.1038/s41598-018-30981-x (PMC6107667; doi:10.1038/s41598-018-30981-x)
Supplement: Supplementary file 1 — Supplementary Information [file 41598_2018_30981_MOESM1_ESM.pdf]

Supplementary Material for: Non-calorimetric determination of absorbed power during magnetic nanoparticle based hyperthermia

I. Gresits,[1] Gy. Thuróczy,[1] O. Sági,[2] B. Gyüre-Garami,[2] B. G. Márkus,[2] and F. Simon[2]

[1]Department of Non-Ionizing Radiation, National Public Health Institute, Budapest
[2]Department of Physics, Budapest University of Technology and Economics and MTA-BME Lendület Spintronics Research Group (PROSPIN), Po. Box 91, H-1521 Budapest, Hungary

PACS numbers:

I. DERIVATION OF THE EXPRESSION FOR THE POWER ABSORBED IN THE SAMPLE

We assume that the resonator which is loaded with the sample is critically coupled, i.e. maximum 1% of the power can be reflected back to the source, or S_{11} is smaller than -20 dB. This incoming power is thus nearly equal to the driving power of the source, i.e. $P_{\text{input}} \sim P_{\text{source}}$. In the absence of added ferrite, the resonator quality factor is Q_{ref} and is filled with water in our simplified case, or with a phantom filled with an appropriate artificial tissue emulating material. In the presence of a ferrite material, the quality factor is lowered to Q_{ferrite} due to the additional loss. The input power dissipation is divided between the resonator (this includes all kinds of losses also due to the solvent in which the ferrite is dissolved) plus the ferrite sample as:

$$P_{\text{input}} = P_{\text{resonator}} + P_{\text{ferrite}}, \tag{1}$$

where $P_{\text{resonator}}$ and P_{ferrite} denote the power dissipated in the resonator and the additional ferrite, respectively. The ratio of the two terms can be obtained from the respective quality factors:

$$\frac{P_{\text{resonator}}}{P_{\text{ferrite}}} = \frac{Q_{\text{ref}}^{-1}}{Q_{\text{ferrite}}^{-1} - Q_{\text{ref}}^{-1}}. \tag{2}$$

Solving these two equations yields:

$$P_{\text{ferrite}} = P_{\text{input}} \left(1 - \frac{Q_{\text{ferrite}}}{Q_{\text{ref}}}\right), \tag{3}$$

We also show in the main manuscript that the resonator quality factor in the presence of the ferrite, Q_{ferrite} can be deduced as:

$$\frac{1}{Q_{\text{ferrite}}} = \frac{1}{Q_{\text{ref}}} + k \cdot Q_{\text{ferrite}}, \tag{4}$$

whose relevant solution is:

$$Q_{\text{ferrite}} = \frac{-1 + \sqrt{1 + 4kQ_{\text{ref}}^2}}{2kQ_{\text{ref}}} \tag{5}$$

This allows to obtain the absorbed power ratio, $a = P_{\text{ferrite}}/P_{\text{input}} = 1 - Q_{\text{ferrite}}/Q_{\text{ref}}$ as

$$a = 1 - \frac{-1 + \sqrt{1 + 4kQ_{\text{ref}}^2}}{2kQ_{\text{ref}}^2}. \tag{6}$$

This function is plotted in the main manuscript. It is clear that this function only depends on the product kQ_{ref}^2, therefore its shape is universal for the resonator loss problem.

II. DETAILS OF THE QUALITY FACTOR MEASUREMENT

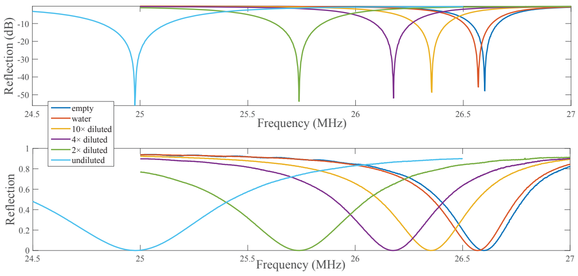

FIG. 1: The reflection curves for resonators which are empty or filled with samples. The upper panel shows the S_{11} reflection parameter on log scale as obtained with the scalar network analyzer. The lower panel shows the reflected power shown on a linear scale. Critical coupling was achieved for all measurements.

Fig. 1. shows the results of reflectometry obtained using a scalar network analyzer and also the same type of data obtained with a power detector. The earlier allows for a more accurate determination of the quality of circuit matching, whereas the latter allows for a better determination of the resonator parameters, quality factor and resonant frequency. In the latter measurement, a computer control also allows for a repeated data acquisition which leads to a good estimate of the mean and variance of these parameters.

III. DETAILS OF THE CALORIMETRIC MEASUREMENTS

Fig. 2. shows a typical time dependent heating curve using the RF irradiation. The apparently linear domain in $T(t)$ allows to determine the $\frac{\mathrm{d}T}{\mathrm{d}t}$ derivative using a linear fit onto the the steepest part of the curve. It is important to mention that there is a lag between the onset of temperature rise with respect to the power turn-on due to the thermal inertia of the sample. The warm up may also cause thermal circulations[1] but this effect is neglected. The RF coil also warms up considerably as we do not employ a cooling. Its effect is, however, minimized by a good thermal isolation between the sample and the coil. In practice, an RF coil for hyperthermal treatment is made of copper tube with an appropriate cooling water flow[2]. When this effect become significant, it may also lead to an unwanted resonator detuning.

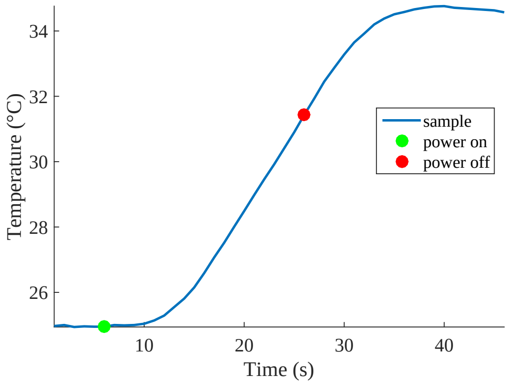

FIG. 2: Temperature increase of the sample (solid curve). Green and red circles indicate the start and the stop time of the RF irradiation.

IV. ADDITIONAL INFORMATION ON THE BIRDCAGE RESONATOR

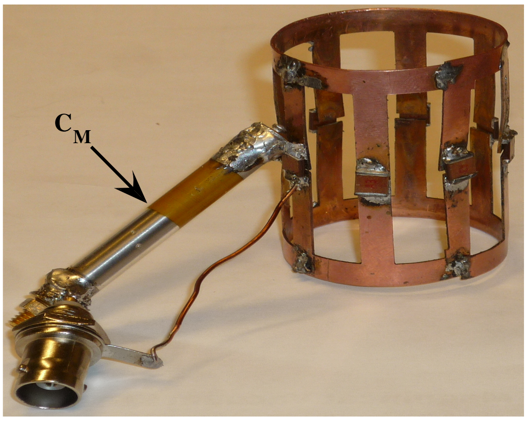

FIG. 3: Photograph of the 8-leg low-pass birdcage coil. A trimmer capacitor acts as circuit matching element.

Fig. 3. shows a photograph of the employed birdcage coil. Note the low-pass construction of the coil, i.e. that capacitors are in the middle of the legs. A trimmer capacitor (Voltronics Inc.) is soldered to the coil which serves for the circuit matching. It is important that the matching trimmer capacitor is soldered as close as possible to the two electrodes of a capacitor as otherwise no perfect matching can be achieved. The birdcage dimensions were designed to allow the irradiation of a laboratory mouse, it has a diameter of 30 mm and length of 50 mm. The coil body is made of copper stripes with 3 mm width and 1 mm thickness. The high-Q RF capacitors have $C = 1000\,\mathrm{pF}$ (type CORNELL DUBILIER - MC22FA102J-F-CAP, 100V) with a variation of $\pm 5\,\%$ of which a few were selected with $\pm 1\,\%$ capacitance variation.

[1] Wang, S. Y., Huang, S. & Borca-Tasciuc, D. A. Potential sources of errors in measuring and evaluating the specific loss power of magnetic nanoparticles in an alternating magnetic field. *IEEE Transactions on Magnetics* **49**, 255–262 (2013).

[2] Garaio, E., Collantes, J. M., Plazaola, F., Garcia, J. A. & Castellanos-Rubio, I. A multifrequency eletromagnetic applicator with an integrated ac magnetometer for magnetic hyperthermia experiments. *Measurement Science and Technology* **25**, 115702 (2014).
